# Evaluation of the optical performance for aspheric intraocular lenses in relation with tilt and decenter errors

**DOI:** 10.1371/journal.pone.0232546

**Published:** 2020-05-04

**Authors:** Jesús Pérez-Gracia, Alejandra Varea, Jorge Ares, Juan A. Vallés, Laura Remón

**Affiliations:** Departamento de Física Aplicada, Universidad de Zaragoza, Zaragoza, Spain; Nicolaus Copernicus University, POLAND

## Abstract

**Purpose:**

To evaluate and compare the effect of misalignment and tilt on the optical performance of different aspheric intraocular lens (IOL) designs.

**Methods:**

Three aspheric IOLs with a different quantity of spherical aberration (SA) have been designed and the effect of IOL misalignment and tilt on the imaging quality of an eye model has been numerically assessed using a commercial optical design software. The prototypes have been manufactured by lathe turning and tested in vitro using the same optical bench (PMTF, Lambda-X) that complies with International Organization for Standardization standard 11979–2 requirements. Image quality was evaluated from the modulation transfer functions (MTFs), through-focus modulation transfer functions (TF-MTFs), root mean square (RMS) values of defocus, astigmatism and coma, and images of the United States Air Force (USAF) target were taken. A comparison with the optical performance of spherical IOLs has also been performed.

**Results:**

Intraocular lens misalignment and tilt increased wavefront aberrations; the effect of misalignment on root mean square (RMS) astigmatism and coma was positively correlated with the spherical aberration of the IOL. Aberration-free IOLs showed the highest MTF for all misalignment values and for IOLs with negative SA correction the MTF decays below 0.43 when they are decentered 0.50 mm.

**Conclusions:**

Aspherical IOLs are more sensitive than spherical IOLs to misalignment or tilt, depending on their SA correction. The optical degradation caused by IOL misalignment had a greater effect on IOL designs with a higher amount of negative spherical aberration. In contrast, the effect of tilt on the optical performance was less sensitive to the IOL design.

## Introduction

Since Harold Ridley implanted the first intraocular lens (IOL) made of poly-methyl-methacrylate (PMMA) in 1949 [1] there have been significant improvements in surgical techniques, material developments [2], accuracy of preoperative measurements [3], and intraocular lens technology [4] in order to achieve complete spectacle independence after cataract surgery. Nowadays, different IOL designs are available in the market [5] such as toric IOLs to correct pre-existing corneal astigmatism, multifocal and accommodative to simultaneously provide good distance and near vision and aspheric IOLs to provide a better optical quality than the traditional IOLs with spherical optical design [6,7]. Aspheric designs are not limited to monofocal lenses; multifocal IOLs, toric IOLs or toric-multifocal IOLs and accommodative configurations may also include an aspheric surface [8].

Typical aspheric IOLs have been designed with negative spherical aberration (SA) to compensate for the average positive SA of the human cornea (approximately 0.27 μm) whereas other designs partially corrected the corneal SA leaving a slightly positive total ocular SA (approximately 0.10 μm) [7,9,10]. The main disadvantage of these IOLs is that the levels of corneal aberration are variable, for example, after myopic refractive correction corneal SA changes to a more positive value, while after hyperopic refractive correction corneal SA changes from a positive to a negative value [11]. In both cases, the negative SA of the IOL may not be beneficial to the patient [12]. Recently, some aspheric IOLs have been designed to be aberration-free, that is, without any spherical aberration. They do not add any aberration to that already existing in the eye and, therefore, it remains with the original corneal SA. The main advantage of this type of lenses is that they are less affected by decentering errors in comparison with negative spherical aberration IOLs [13].

The biomechanical stability inside the capsular bag is the key feature leading to a successful surgical procedure with this type of lenses. Misalignment or tilt can affect the optical performance inducing wavefront aberrations that may result in significant visual disturbances [14–16]. On the one hand, there are some clinical studies [17–19] that measure the IOL misalignment, tilt or rotation once it has been implanted inside a pseudophakic eye using different measurement methods such as Scheimplug imaging, Purkinje reflections [20], optical coherence tomography [19], and slit lamp assessment [17]. However, the heterogeneity of experimental methods and the fact that only one IOL model per eye is considered make results comparison difficult. On the other hand, the impact of IOL misalignment and tilt on imaging quality has been evaluated in theoretical and in-vitro studies and the dependence on the aspheric IOL design of the effect of misalignment on optical quality has been established [6,10,13]. Eppig et al. [6] evaluated the effect of aspheric IOL misalignment and tilt on the imaging quality using computer simulations in a schematic eye model assuming geometrical optics. Finally, other studies evaluated experimentally the optical performance of different currently marketed aspheric monofocal IOLs and its degradation with IOL misalignment and tilt [10,13]. However, there are several commercial aspheric IOLs that are designed and manufactured for a specific cornea making comparison difficult between the different designs.

The objective of this study is to accomplish a comprehensive analysis of the image quality offered by aspheric IOLs and to compare it with the performance of spherical designs. Therefore, aspheric IOLs with different amounts of SA have been designed and the effect of IOL misalignment and tilt on the imaging quality of an eye model has been assessed. Moreover, the aspheric designs have been fabricated and evaluated using a PMTF system. Finally, a comparison between theoretical and experimental results is presented.

## Material and methods

### Eye model

In order to design the aspheric IOLs and to evaluate the effect of misalignment and tilt on their optical performance a numerical model of a pseudophakic eye was implemented with a commercial optical design software (OSLO EDU Edition 2001–2012, Revision 6.6.0 –Lambda Research Corporation) and ray-tracing simulations were performed. The model was based on the Navarro schematic eye [21] (see Table 1 for details) for far vision, where the eye lens has been replaced with a particular IOL. The initial position of the IOL within the pseudophakic eye model was set so that its anterior principal plane was at the same position as the anterior principal plane of the crystalline lens of the original phakic eye. The cornea of the eye model has a refractive power of 42.16 diopters (D) and a fourth-order Zernike ($Z_{0}^{4}$) standard spherical aberration of 0.139 μm for a 6.00 mm entrance pupil diameter (5.51 mm iris diameter). For each design, the vitreous camera depth was set in order to get the paraxial image in retina for each corresponding IOL power.

**Table 1:** Eye model and parameters used for designs and simulations.

Medium	Radius (mm)	Thickness (mm)	Refractive index at 555 nm	Conic Constant
**Anterior surface of cornea**	7.72	0.55	1.376	-0.26
**Posterior cornea**	6.50	2.46	1.336	0
**Pupil**	Infinite	To be determined according to IOL design	1.336	----
**IOL´s anterior surface**	To be determined according to IOL design	To be determined according to IOL design*	1.485	Depending on the type of aspherical surface.
**IOL´s posterior surface**	To be determined according to IOL design	To be determined according to IOL design**	1.336	0
**Retina**	-12.00			0

* Distance between vertex of the posterior cornea and the vertex of the anterior surface of the IOL is 5.07 mm, 5.00 mm, and 4.94 mm for refractive powers of +10.00 D, +20.00 D and +35.00 D, respectively.

**Distance between vertex of the posterior surface of the IOL and retina is 21.03 mm, 17.91 mm, and 14.57 mm for refractive powers of +10.00 D, +20.00 D and +35.00 D, respectively.

### Intraocular lens designs and construction

Several IOLs models with refractive powers +10.00 D, +20.00 D and +35.00 D were designed for a semi-finished (i.e. the radius for the back surface is previously determined by the manufacturer) hydrophobic acrylic material (Benz HF-1.2 Universal Blank) with a refractive index n = 1.485 at the design wavelength λ_0_ = 546 nm.

For each refractive power, two types of aspherical IOL with anterior conical surface were designed. The aspheric surface used to model the different IOLs takes the form of a rotationally symmetric conic cross section with the sagita defined as:

$$z(r)=\frac{{cr}^{2}}{1+\sqrt{1-(1+k)c^{2}r^{2}}}$$ (1)

where *z* is the sagita, *r* is the radial coordinate surface, *c* is the vertex curvature, and *k* is the conic constant. For -*1<k*<0, the curvature becomes increasingly flatter at the periphery, *k* = 0 is a sphere, and for *k*>0 the curvature becomes steeper at the periphery. Table 2 shows the IOL design parameters used for the simulation. Lens A was an IOL with fourth-order Zernike negative SA to totally compensate for the fourth-order Zernike positive SA of the Navarro cornea and Lens B was designed as an IOL that does not add any fourth-order Zernike SA to the eye. Futhermore, for refractive power of +20.00 D, an additional Lens C was designed using Eq 1 with an amount of SA to partially correct the positive fourth-order Zernike SA of Navarro’s cornea. In this last case, the total ocular SA is slightly positive (+0.069 μm). To perform the optimization a commercial optical design software (OSLO) was used and the fourth-order Zernike spherical aberration was utilized to determine the suitable conic constant. The fourth-order Zernike coefficient ($Z_{0}^{4}$) was expressed according to the American National Standards Institute Z80.28–2017 [22]. Table 2 shows the spherical aberration of IOL for 6.00 mm pupil diameter. For comparison, spherical IOLs with the same dioptric powers and radius of curvature were designed and evaluated.

**Table 2:** Parameters of IOL lenses used in the simulations and experiments. *K* represents the conic constant values and SA represents the spherical aberration of IOL for 6.00 mm pupil diameter.

IOLs	Radius Curvature (mm)		Center Thickness (mm)*		K	SA (μm) Ø 6 mm
	Anterior	Posterior				
**+10.00 D**	44.56	-22.35	0.65	**Lens A**	-1600	-0.139
				**Lens B**	-101.53	0.000
				**Spherical**	0.00	+0.05
**+20.00 D**	15.89	-13.94	0.96	**Lens A**	-30.47	-0.139
				**Lens B**	-10.91	0.000
				**Lens C**	-19.36	-0.069
				**Spherical**	0.00	+0.123
**+35.00 D**	7.76	-9.27	1.26	**Lens A**	-5.41	-0.139
				**Lens B**	-3.36	0.000
				**Spherical**	0.00	+0.328

*The center thickness was calculated to get 0.35 mm edge thickness at the full diameter of 6.00 mm.

Finally, all spherical and aspherical IOLs designs were manufactured following a lathe-milling process using Optoform 40 lathe (Sterling Ultra Precision, USA) by AJL Ophthalmic S.A. (Spain). Three different IOLs of each model were manufactured. Differences between the theoretical design and the manufactured IOL profiles were lower than 0.1 mm as measured with a contact profilometer (DEKTA kXT, Bruker).

### Numerical simulations

Once each IOL was designed, its optical performance was evaluated inside the eye model using OSLO optical design software for different alignment conditions. First, the IOLs were decentered in horizontal direction from 0.00 mm (on axis) to 1.00 mm in 0.25 mm steps relative to the pupil center. Secondly, the optical IOLs axis was tilted relative to the corneal optical axis with vertex in the pupil center (from 0.00 degree to 5.00 degree in steps of 1.00 degree). For each misalignment and tilt, tangential and sagittal Modulation Transfer Function (MTF) at 100 cycles per degree for a 3.00 mm pupil diameter were calculated following the procedure described in the ISO 11979–2 [23]. Zernike wavefront aberration coefficients associated with defocus ($Z_{0}^{2}$), astigmatism ($Z_{2}^{-2}$ and $Z_{2}^{2}$), and primary coma ($Z_{3}^{-1}$ and $Z_{3}^{1}$) were computed for a wavelength of 546 nm. The root mean square (RMS) was calculated as $Z_{0}^{2}$ and the square root of the sum ($Z_{2}^{-2}$ and $Z_{2}^{2}$) or ($Z_{3}^{-1}$ and $Z_{3}^{1}$) squared for defocus, astigmatism and primary coma, respectively. The optical performance estimation was carried out for all the IOLs under consideration.

### Experimental measurements

The optical performance for +20.00 D IOLs was experimentally tested in vitro with the PMTF optical bench (Lambda-X, Belgium). This device follows the requirements of ISO standards 11979–2 [23]. To obtain the MTF information, the set up uses the slanted edge technique, which consists in imaging an edge onto the detector. To measure the optical quality of an IOL, a model cornea with +0.280 μm SA for a 5.00 mm pupil aperture was used following ISO specifications.

**Fig 1:**
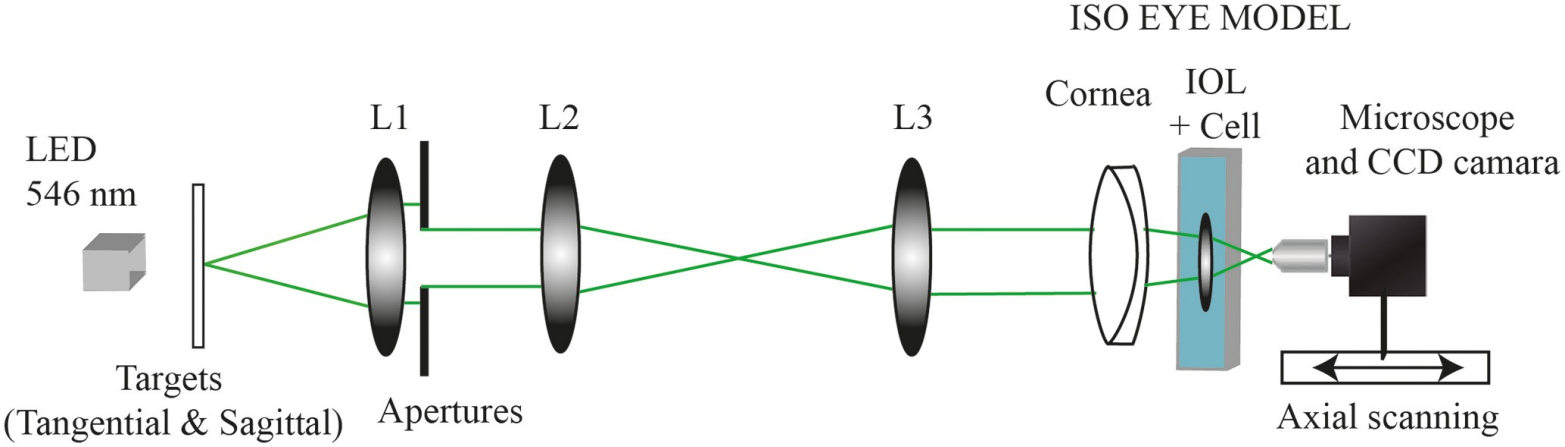
Schematic illustration of the experimental set up (PMTF) used to make the experimental measurements.

A schematic illustration of the experimental setup is shown in Fig 1. The illumination system consists of a LED lamp that emits at 546 nm. This source, which can be set up as a horizontal or a vertical linear source, is collimated by lens L1, L2 and L3. The artificial eye consists of an artificial cornea and the IOL to be tested is submerged in a wet cell with balanced salt solution (BSS Distra-Sol, Ophcon). The lens is placed inside an 11.0 mm diameter holder that allows controlling misalignments and tilts. A CCD camera, with a pixel size of 4.65 x 4.65 μm attached to an X20 microscope objective (in order to achieve a spatial effective resolution of 0.23 μm/pixel), was used to capture the images formed along the optical axis of the opto-mechanical eye model with the IOL under test.

In this work, the MTF at 100 cycles per degree with a 3.00 mm aperture pupil diameter for different misalignments (0.00 mm to 1.00 mm in steps of 0.25 mm) and tilts (from 0.00 degree to 5.00 degree in steps of 1.00 degree) was measured only for +20.00 D IOLs. The IOLs were tilted first and then decentered, in the specific case of measuring optical quality in a tilt-misalignment combination (4° tilt and 0.75 mm misalignment). For experimental results and due to the fact that MTF function loses axis symmetry when the IOLs are decentered and tilted, MTF values have been calculated as the average of tangential and sagittal MTF values. To simulate the different tilt conditions, first, a customized aluminum wedge was inserted under the cuvette floor to tilt the IOL axis in a controlled way with respect to the optical axis of the PMTF. Secondly, the IOL center was aligned in relation to the pupil center of the PMTF using the Siemens star target. Controlled lens decentering was achieved by means of the calibrated displacement of the PMTF X-Y translation table. In addition, images of the 1951 USAF target through the artificial eye were registered at the best focus for different misalignments, tilts and one combination of misalignment and tilt and evaluated. The software automatically locates the best focus at 50 line pairs/mm because the CCD camera can be moved along a rail, thus providing the peak signal intensity for each position using a through-focus algorithm. Finally, the monochromatic through-focus MTF (TF-MTF) at a 3.0 mm pupil size and 100 cycles per degree was also measured, with an axial scanning from 18 to 22 diopters.

## Results

### Numerical results

**Fig 2:**
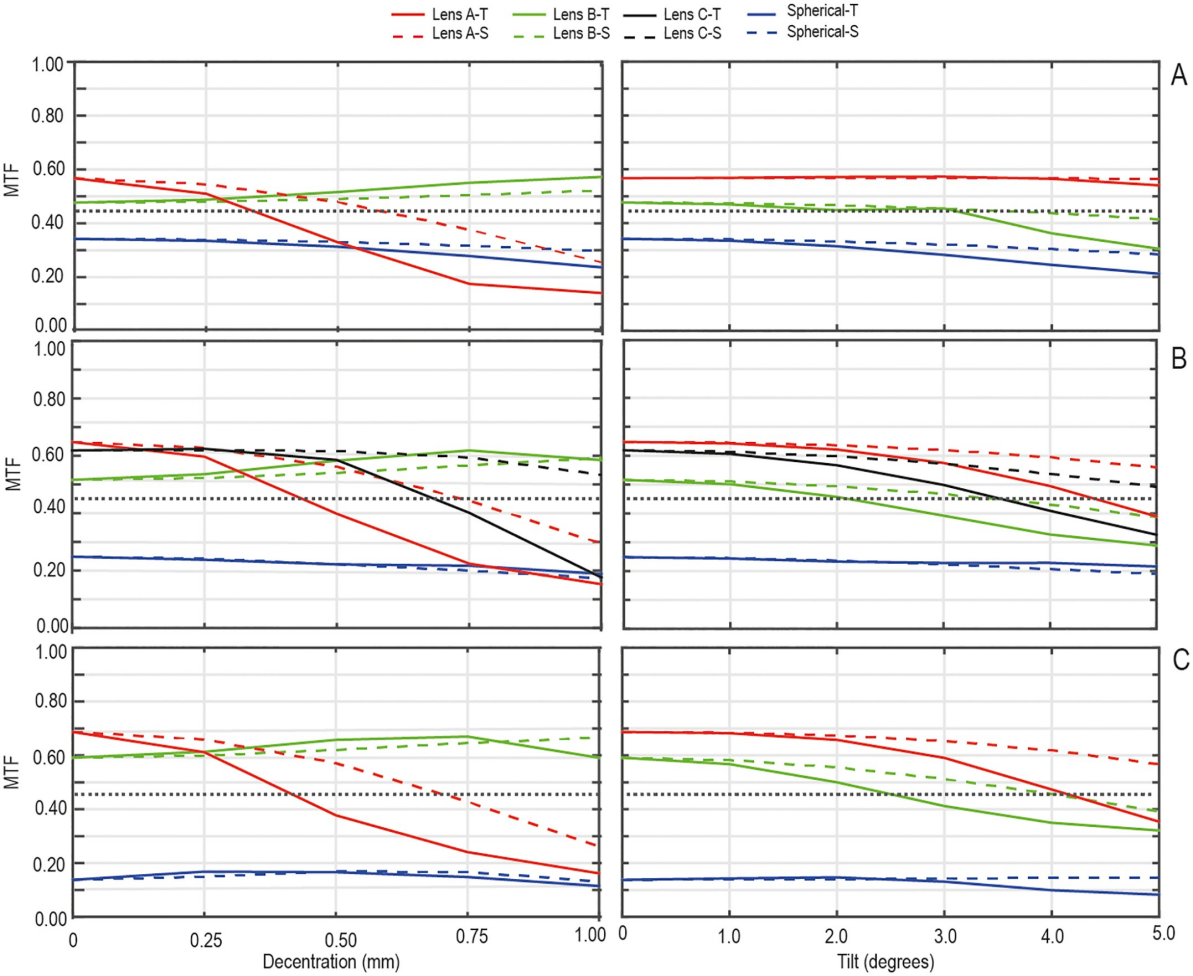
The MTF of the tested IOLs as a function of decentration (left column) and tilt (right column) with a 3.00 mm pupil diameter and 100 cycles per degree. The tangential MTF (continuous line) and the sagittal MTF (dashed line) are shown for each refractive power: A) +10.00 D, B) +20.00 D, and C) +35.00 D. The horizontal dashed line represents the value of MTF 0.43 specified in the ISO 11979–2 [23].

In order to determine the optical performance of the different designs, on–axis and tangential and sagittal MTF were measured for each misalignment and tilt at 100 cycles per degree with a 3.00 mm diameter pupil following the procedure described in the ISO 11979–2. Fig 2 shows on-axis MTFs and tangential and sagittal MTF results for misalignment (left column) and tilt (right column). Fig 2a, 2b and 2c show the MTF for the IOLs with a refractive power of +10.00 D, +20.00 D, and +35.00 D, respectively. For comparison, the MTFs for the respective spherical IOLs are plotted in the same figure. Additionally, Table 3 shows the misalignment or tilt values for which the MTF is below 0.43, the tolerance limit specified in the ISO 11979–2 [23]. In what regards on-axis MTF, in Fig 2 (left column) it can be seen that spherical IOLs clearly performed the worst for all refractive powers, their MTF values being almost misalignment independent. For the three refractive powers Lens A on-axis MTFs are slightly higher than those of Lens B or Lens C. However, when the IOL was decentered the amount of MTF degradation was strongly dependent on the IOL design. Lens A MTFs rapidly decay with misalignment and when decentered 0.50 mm, its MTF value decays below 0.43 for all refractive powers. Lens B was scarcely sensitive to misalignment for all refractive powers, even the MTF value increased with misalignment. Lens B MTF never decays below 0.43 for any misalignment. Lens C offers better on-axis MTF values than Lens B below 0.50 mm misalignment. However, when decentered 0.75 mm (see Table 3), its MTF value falls below 0.43.

**Table 3:** Minimum values of misalignment and tilt for which MTF is below 0.43 (for 100 cycles per degree and a 3.00 mm diameter pupil).

IOL design	Lens A			Lens B			Lens C
Refractive power (D)	10.00	20.00	35.00	10.00	20.00	35.00	20.00
Misalignment (mm)	0.50	0.50	0.50	-----	-----	-----	0.75
Tilt (degrees)	-----	-----	5.00	4.00	3.00	3.00	5.00

“-----”means MTF never goes under 0.43 in the analyzed misalignment and tilt ranges.

Fig 2 (right column) shows MTF as a function of tilt. In this case, the MTF was less sensitive to the IOL design than for the IOL misalignment. For the three simulated refractive powers, the spherical IOL MTF was almost tilt independent. In what regards aspherical designs, or +10.00 D, Lens A provided almost constant MTF over a wide range of tilt whereas Lens B MTF goes below 0.43 for 4.00 °. For +20.00 D, MTF decays slowly with tilt for all IOL designs and Lens B and C presented a MTF below 0.43 for tilts over 4.00° and 5.00° respectively (see Table 3). Finally, for +35.00 D, Lens A had a MTF below to 0.43 for a 5.00° tilt while for Lens B the tilt value was 3.00°.

**Fig 3:**
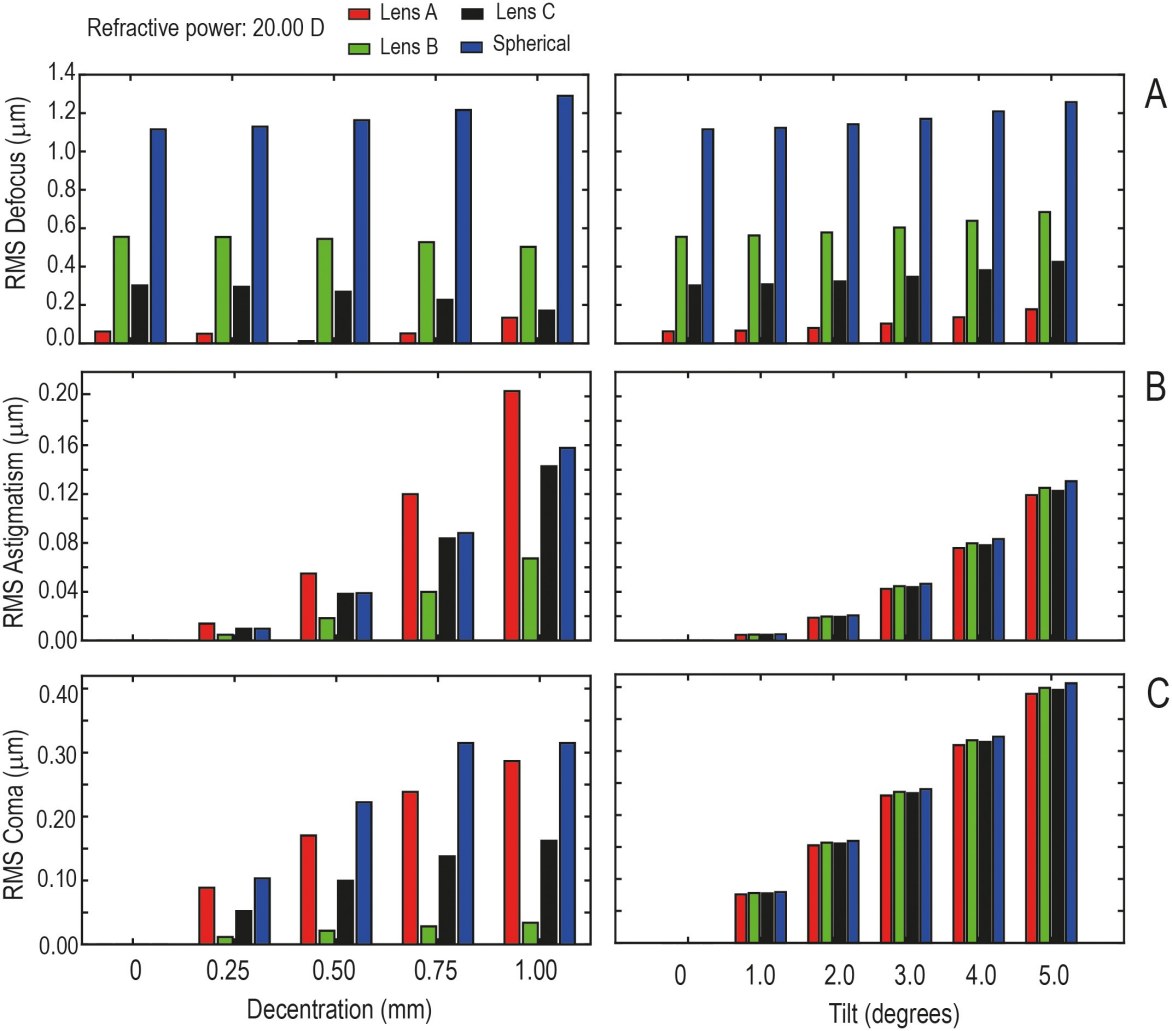
Theoretical results of RMS for decentration (left column) and for tilt (right column) for a refractive power of +20.00 D and a 6.00 mm diameter pupil: A) RMS defocus, B) RMS astigmatism and C) RMS coma.

From the Zernike wavefront aberration coefficients associated with defocus ($Z_{0}^{2}$), astigmatism ($Z_{2}^{-2}$ and $Z_{2}^{2}$), and primary coma ($Z_{3}^{-1}$ and $Z_{3}^{1}$), the RMS was calculated for a 3.00 mm pupil diameter for all the designed IOLs. Fig 3 shows the RMS for misalignment (left column) and for tilt (right column) for a refractive power of +20.00 D. Fig 3a), 3b) and 3c) show the RMS defocus, the RMS astigmatism and the RMS coma, respectively. In general, it can be seen that IOL misalignment and tilt increase wavefront aberrations regardless of the IOL design. As it can be expected, without misalignment and tilt, RMS astigmatism and coma are negligible. Clearly, spherical IOLs induce the highest value of RMS defocus and for Lens B it was higher than for Lens A and C for any misalignment and tilt. The effect of misalignment on RMS astigmatism and coma was positively correlated with the spherical aberration of the IOL. According to this fact, Lens A presents higher RMS values of astigmatism and coma than the other IOL designs. On the contrary, the effect of tilt on RMS astigmatism and coma was less influenced by the IOL design.

### Experimental results

**Fig 4:**
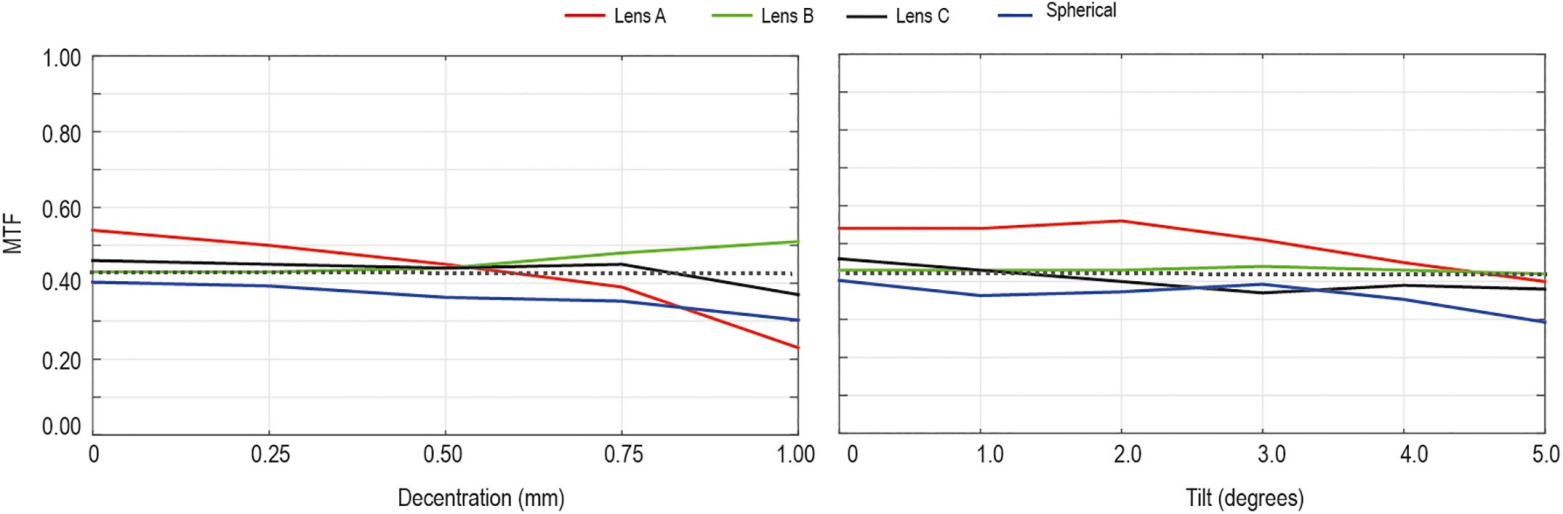
**The experimental MTF (3.00 mm pupil diameter and 100 cycles per degree) for a refractive power of +20.00 D tested in-vitro with the PMTF optical bench as a function of: Left column: Decentration**. Right column: tilt.

Fig 4 shows the experimental MTF (average between tangential and sagittal MTF values) results for misalignment (left column) and tilt (right column) for a refractive power of +20.00 D. As it was predicted by numerical calculation (Fig 2), the MTF degradation was dependent on the IOL design, being more dependent on misalignment than on tilt. Lens A on-axis MTFs are slightly higher than those of Lens B, Lens C or spherical lenses. Lens A is the most affected by misalignment, its MTF rapidly decays, so that when decentered 0.50 mm the MTF value is lower than 0.43. Lens B MTF never decays below 0.43 for any misalignment and for Lens C MTF value is below 0.43 when it is decentered 0.75 mm. Regarding to tilt effect, the MTF was almost tilt independent for the different designs. For tilts over 5.00° and 3.00° Lens A and Lens C had a MTF below 0.43, respectively, but for model B MTF never diminishes below 0.43 for any tilt. These results are similar to those obtained numerically.

**Fig 5:**
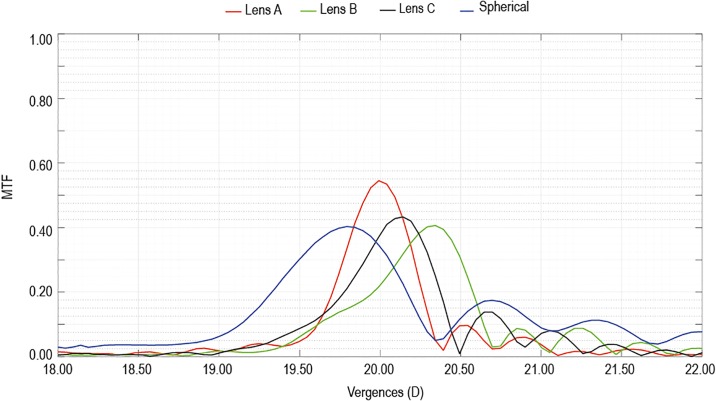
Experimental TF-MTF (3.00 mm pupil diameter and 100 cycles per degree) for a refractive power of +20.00 D tested in-vitro with the PMTF optical bench.

Fig 5 shows the experimental TF-MTF for 100 cycles per degree obtained in the PMTF for a 3.00 mm diameter pupil and 546 nm wavelength. As it can be seen, on the one hand the MTF for Lens A is higher than for Lens B, C or spherical IOLs designs. On the other hand, the other lenses have a longer depth of focus than Lens A.

**Fig 6:**
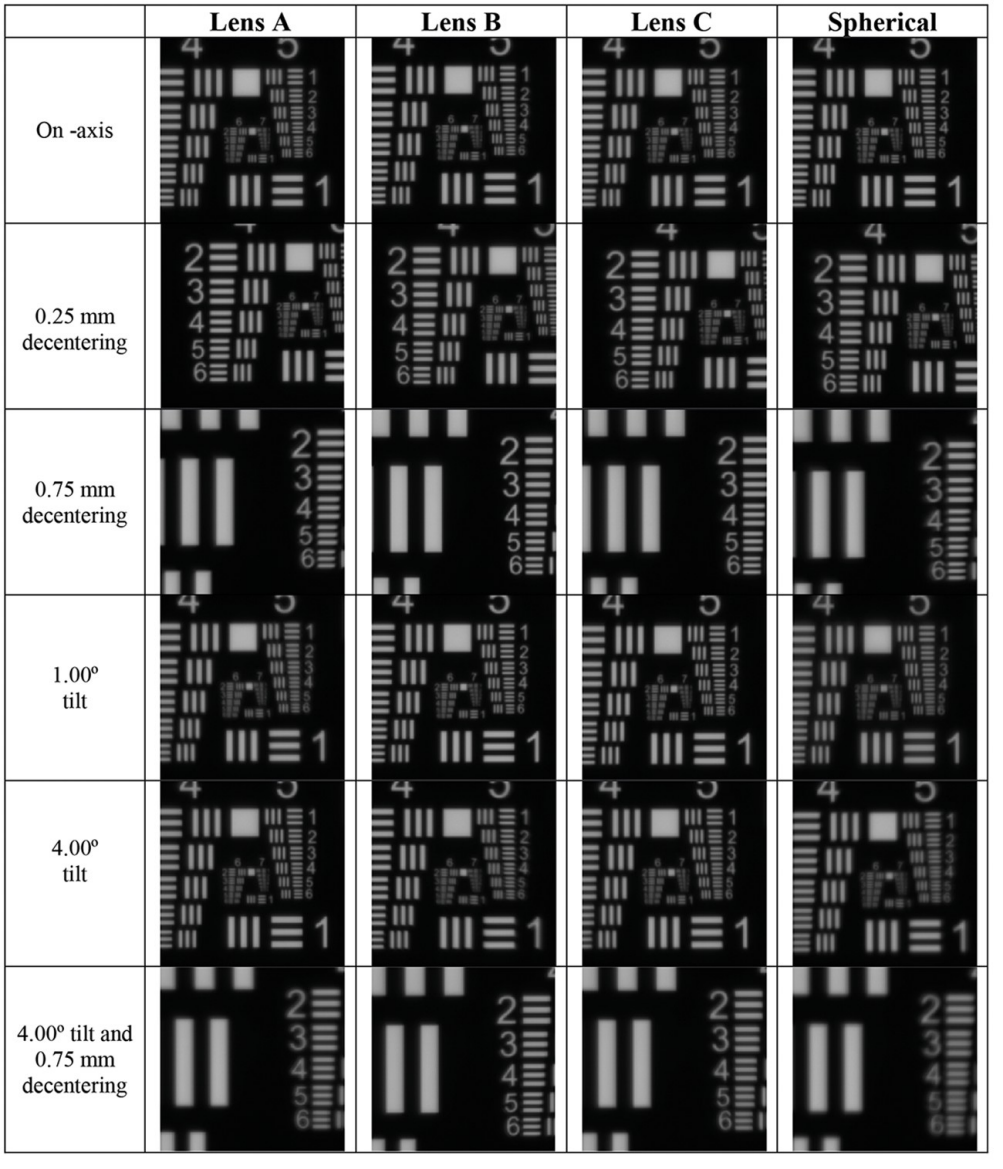
Experimental 1951 USAF test images for a refractive power of +20.00 D and for all the IOL designs. Images on-axis, for misalignments of 0.25 and 0.75 mm, tilts of 1° and 4° and a combination of 0.75 mm of misalignment and 4° of tilt are shown.

Fig 6 shows the images of the 1951 USAF target with a 3.00 mm diameter pupil for a refractive power of +20.00 D and for all the IOL designs. The images were taken at the best focus, with misalignments of 0.25 and 0.75 mm, tilts of 1° and 4° and a combination of 0.75 mm of misalignment and 4° of tilt. Agreeing with the theoretical results, IOL decentration effect is more dependent on the IOL design than tilt’s. As we mentioned above, Lens A and spherical IOL is more sensitive to misalignment than Lens B and Lens C (see 3^rd^ row in Fig 6).

## Discussion

Postoperative IOL stability is a decisive feature in order to ensure a successful surgical procedure. The stability of the IOL inside the capsular bag depends on some factors, including IOL diameter versus bag diameter, surgical technique, material properties and haptic design [24]. For premium IOLs such as aspheric, toric, multifocal or accomodative, the final position of the IOL is more critical [6,7] than for spherical ones to ensure the optimal optical performance.

In this work, we designed and manufactured different aspheric IOLs and evaluated the effect of IOL misalignment and tilt on the optical performance both theoretically (on an eye model) and experimentally (using the PMTF system). Calculations were performed with monochromatic green light (546 nm wavelength) using commercial optical design software and the theoretical Navarro eye model, where the crystalline lens was replaced with a particular IOL. The sagittal and tangential MTF at 100 cycles per degree for a 3.00 mm diameter pupil were calculated and the RMS was computed from the different aberration coefficients. The experimental measurements were performed with a PMTF system following the specifications described by the ISO 11979–2 [23]. In addition, with the same device, images of the 1951 USAF target were taken at the best focus for different misalignments, tilts and a combination of misalignment and tilt. The TF-MTFs for different defocused planes were registered. The study considered 5 misalignment values (0.00 mm (on axis) to 1.00 mm; a 0.25 mm step) and 6 tilt values (0.00 degrees (on axis) to 5.00 degrees; a 1.00 degree step), which are similar to those reported in clinical studies [2,25–27]. The typical mean misalignment in clinical studies is 0.30±0.16 mm [a range from 0.00 mm to 1.09 mm] and the mean tilt is 2.62±1.14 degrees [a range from 0.20 to 8.17 degrees].

To our knowledge, this is the first study where different aspheric IOLs designs with different amount of SA for a specific model cornea have been designed and manufactured, and their optical quality has been both numerically and experimentally determined as a function of IOL misalignment and tilt. Eppig et al. [6] only evaluated the effects of misalignment and tilt on the MTF and the combination of misalignment and tilt was not evaluated. Lawu et al. [13] evaluated the wavefront aberrations and the retinal visual images (Landolt Rings).

We found numerically and experimentally that the optical quality (in terms of MTF) is dependent on the amount of spherical aberration correction (see Figs 2 and 4). However, the effect of IOL tilt was less sensitive to the IOL design than the effect of IOL misalignment. Compared to the aspherical IOL models, spherical IOLs provide a worse MTF performance in perfectly centered and non-tilted positions. For the three refractive powers Lens A on-axis MTF is slightly higher than those of Lens B or Lens C. However, when the IOL was decentered the amount of MTF degradation was strongly dependent on the IOL design. Lens A is more sensitive to misalignment than Lens B and Lens C. For Lens B and spherical IOLs MTFs were almost misalignment independent. These findings are in good agreement with results in previous experimental [6,10,13,28,29] and clinical studies [12, 30, 31] that showed that the effect of tilt was not sensitive to the IOL design for a similar tilt range. Oppositely, the effect of IOL misalignment was sensitive to the IOL design, being stronger for IOLs designs with higher SA values. The experimental USAF images in Fig 6 are consistent with the numerical and experimental MTF results. The combined effect of misalignment and tilt depends on the IOL designs, Lens A and spherical lens are the models that were more affected in this situation.

In relation with the ISO quality standard regarding image quality, as it can be seen in Table 3, tolerance levels for Lens A require it to be decentered less than 0.5 mm in order to provide a value of MTF (at 100 cycles per degree) higher than the 0.43 specified by the ISO. For the lens C the value of misalignment that degradates the optical performance is 0.75 mm and for Lens B even a 1.00 mm misalignment is well tolerated. These findings are in good agreement with clinical findings reported in several studies. Ale et al. [32] reported that, in average, 2–3 degrees of tilt and a 0.2–0.3 mm misalignment are common, without affecting visual performance. Holladay [9] demonstrated that aspherical lenses can undergo a misalignment of up to 0.4 mm and a tilt of up to 7° and Piers’ studies [27] revealed a higher tolerance to a wrong position, the resulting threshold (critical) values being at 0.8mm of misalignment and 10° of tilt before they start to exhibit a worse performance than their spherical counterparts.

Our results show that IOL misalignment and tilt (see Fig 3) increase wavefront aberrations. In general, RMS defocus, astigmatism and coma increase with decentering, depending on the dioptric power. On the contrary, the effect of tilt on RMS astigmatism and coma was little dependent on the IOL design. As we expected, without misalignment and tilt, RMS astigmatism and coma are negligible. The spherical IOLs cause the highest value of RMS defocus and for Lens B the RMS defocus was higher than for Lens A and C for any misalignment and tilt. Our results agree with the results obtained by Lawu et. al.[13].

As it can be seen in Fig 5, there is a quite good agreement between the behavior of the TF-MTF curves in a centered and untilted position and the corresponding defocus wavefront aberration RMS calculated for this condition (see Fig 3A). The smaller the Zernike defocus RMS, the smaller the displacement of the peak from the TF-MTF curve. The displacement is clearly a minimum for the Lens A. In addition, a similar good agreement can be observed in relation to the residual spherical aberration. As it can be seen, the higher the residual spherical aberration of the combination IOL+Cornea the higher the depth of focus of the system. As it was previously reported [33] this behavior can be interesting to increase the depth-of-field for the IOL user.

In conclusion, it was seen that, regarding image MTF analysis in centered position, aspherical IOLs provide better optical performance than spherical IOLs. However, aspherical IOLs are more sensitive to misalignment or tilt, depending on the aberration correction. The optical degradation caused by IOL misalignment had a greater effect on IOL designs with a higher amount of negative spherical aberration. In contrast, the effect of tilt on optical performance was less sensitive to the IOL design in the clinically typical range of tilt errors evaluated. The obtained results suggest that if the alignment cannot be guaranteed, aberration-free IOLs may be the best option for a specific patient in order to provide an acceptable imaging even with some misalignment and tilt. In this work the aspherical surface was optimized on the IOL’s anterior side. Furthermore, it is important to remark that geometrical aberration it is not the only source that it is going to decrease the image quality when an IOL is decentered. Transversal chromatic aberration [34, 35] is also a potential source of image quality degradation which was not taken into account in this work. However, due to the fact that the main nature of chromatic aberration in a refractive element comes from the material composition, it is not expected any significant differential image performance between our spherical or aspherical tested IOL designs. Further studies may involve other designs with both anterior and posterior aspherics. IOLs were designed for a specific cornea with a determined amount of spherical aberration. In the future, it can be interesting to evaluate these designs in other corneal models such as after myopic correction with laser ablation in order to determine the effect of misalignment or tilt on the optical performance.

## Supporting information

S1 File Theoretical MTF for decentration and tilt. (XLSX)

S2 File Theoretical results of RMS for decentration and for tilt. (XLSX)

S3 File Experimental results of MTF for decentration and for tilt (refractive power: 20.00 D). (XLSX)

S4 File Experimental TF-MTF (3.00 mm pupil diameter and 100 cycles per degree) (refractive power: 20.00 D). (XLSX)
